# Iatrogenic Esophageal Perforation in Extremely Premature Babies With Low Birth Weight: A Case Series at a Single Tertiary-Care Center

**DOI:** 10.7759/cureus.94197

**Published:** 2025-10-09

**Authors:** Saud A Aljadaan, Nawaf S Alharbi, Mohammed K Alnamshan, Suliaman M Alaqeel, Ali O Abaas

**Affiliations:** 1 Pediatric Surgery, King Abdulaziz Medical City, Riyadh, SAU; 2 Pediatric Surgery, King Abdullah Specialist Children Hospital, Riyadh, SAU; 3 Pediatric Surgery, Ministry of the National Guard Health Affairs, Riyadh, SAU

**Keywords:** esophagus, iatrogenic, neonate, perforation, prematurity

## Abstract

Background: Iatrogenic esophageal perforation (EP) is a rare but serious complication of nasogastric tube (NGT) or orogastric tube (OGT) insertion in neonates. Early recognition of this condition is often challenging, as clinical and radiological findings may mimic esophageal atresia. Reported mortality is high, largely owing to the sequelae of extreme prematurity and associated comorbidities. The purpose of this study was to review cases of iatrogenic EP in extremely premature babies with low birth weight and to describe the presentation, management, and outcomes.

Methods: Retrospective chart review of seven patients with iatrogenic EP treated non-operatively at King Abdulaziz Medical City in Riyadh, Saudi Arabia, from 2004 to 2024.

Result: Seven extremely premature infants with iatrogenic EP were managed conservatively without primary surgical repair. All resumed enteral feeding successfully after a mean of 22 days, with no complications directly attributed to the perforation. However, four infants died from comorbidities of extreme prematurity rather than the perforation itself.

Conclusion: Iatrogenic EP diagnosis can be difficult in extremely premature babies with low birth weight. Non-operative management of such cases can be considered a safe and efficient option with no need for subjecting the patient to operative options.

## Introduction

Inserting nasogastric tube (NGT) or orogastric tube (OGT) is a routine practice in the neonatal ICU (NICU), although it may cause rare complications, such as iatrogenic esophageal perforation (EP), especially in certain patient populations [1]. Neonates with low birth weight or extremely premature are at greater risk of developing such complications. Moreover, the recognition of complications of enteric tube placement can be challenging because many affected neonates are extremely premature, have low birth weights and are frequently intubated, making it difficult to distinguish EP from esophageal atresia based on clinical presentation and basic radiological imaging findings [2-4].

Iatrogenic EP increases neonate mortality and morbidity by up to 30% [5]. However, most deaths in such patients are related to sequalae of extreme prematurity, such as cardiopulmonary congenital diseases, sepsis, and necrotizing enterocolitis (NEC) [5,6]. In this study, we reviewed and examined cases of iatrogenic EP in terms of diagnosis, risk factors, management techniques, and outcomes over the last 20 years in a single tertiary-care center.

## Materials and methods

Methodology

The study was conducted as a retrospective chart review over a six-month period, examining seven patients diagnosed with iatrogenic EP at King Abdulaziz Medical City in Riyadh between January 1, 2004, and December 31, 2024.

The inclusion criteria comprised all cases of iatrogenic EP in neonates with a birth weight of less than 1,000 grams and a gestational age of less than 28 weeks, classified as extremely premature. Cases involving neonates with a birth weight greater than 1,000 grams or a gestational age exceeding 28 weeks were excluded from the study.

The diagnostic criteria included difficulty in passing an OGT, chest X-ray (CXR) showing new-onset pneumomediastinum or pneumothorax (PT), and the absence of occlusion of esophagus by esophageal distal gas in stomach on initial radiological imaging.

The chart review was conducted electronically between August 1, 2024, and October 31, 2024. Patients' demographics, diagnostic modalities results, treatment, and outcomes were recorded electronically into an Excel sheet. Data were abstracted into a standardized data collection form and then entered into a secure, password-protected database Excel. Only study investigators had access. Continuous variables were summarized as means, medians with interquartile ranges and categorical variables as frequencies and percentages.

The study received ethical approval from the King Abdullah International Medical Research Center (KAIMRC) under IRB Approval Number 0000024524.

## Results

The cases of seven neonates with iatrogenic EP from 2004 to 2024 were included in this series. All patients were preterm, with an average gestational age of 24.5 ± 2.5 weeks. The mean birth weight was 650 ± 190 grams.

In our series, the diagnostic criteria included difficulty in passing an OGT, CXR showing new-onset pneumomediastinum or PT, and the absence of occlusion of esophagus by esophageal distal gas in stomach on initial radiological imaging. All patients included in our series had an initial presentation of difficult enteric tube placement with suspicion of esophageal atresia. A bedside water-soluble contrast radiological study was performed to assess for leakage into the pleural cavity or mediastinum in only three cases. The diagnosis of iatrogenic EP was confirmed within the first week of life, with a mean age at diagnosis of 4 ± 3 days.

**Figure 1:**
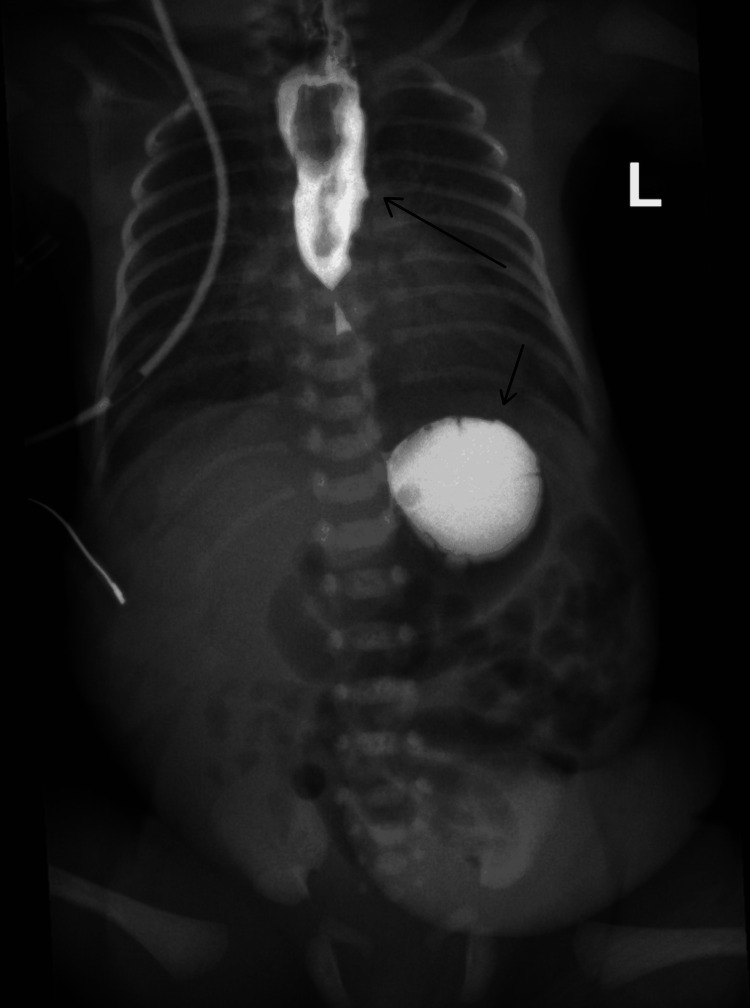
Contrast-enhanced X-ray showing contrast extravasation into the mediastinum causing a widened mediastinum X-ray with contrast injected through the OGT demonstrates proximal esophageal dilatation extending to the mid-esophagus (long arrow) and contrast material visualized within the gastric fundus (short arrow). OGT: Orogastric tube

**Figure 2:**
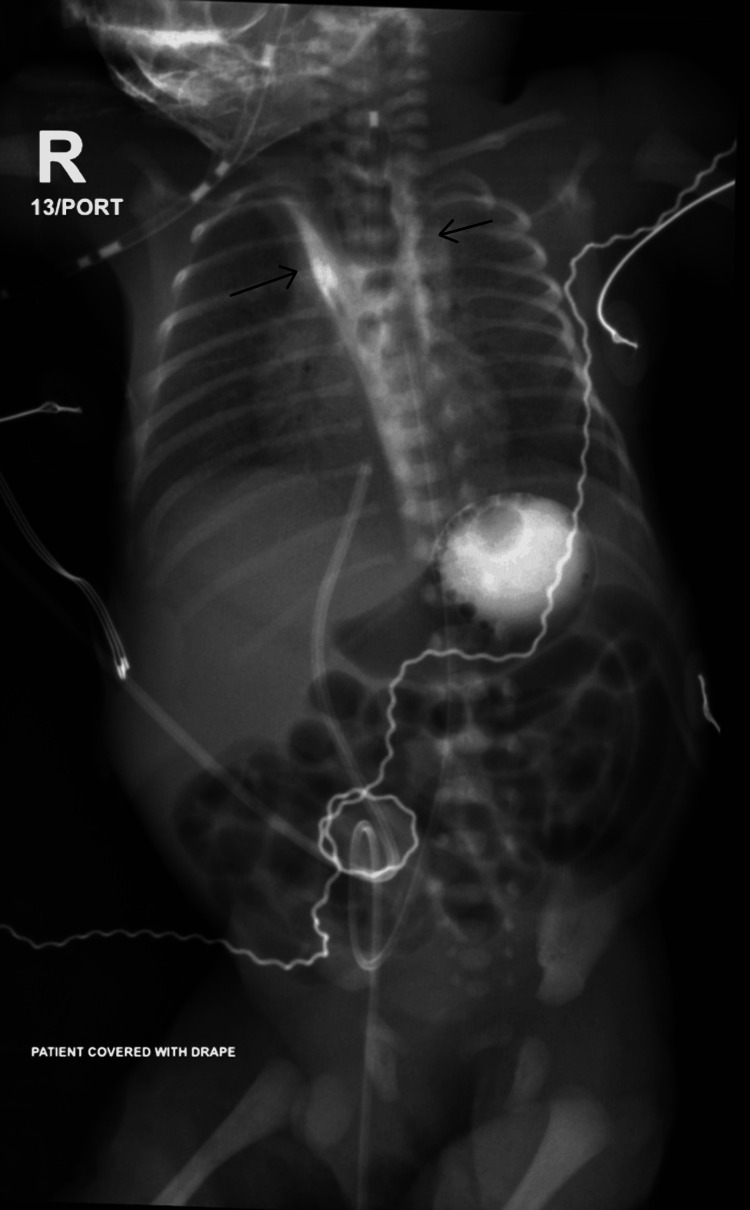
Contrast-enhanced X-ray showing contrast leak into the mediastinum suggestive of EP Contrast-enhanced X-ray shows contrast material within the mediastinum with contrast extravasation into the mediastinal space causing wide mediastinum (the two arrows). EP: Esophageal perforation

The etiology in all cases was confirmed to be iatrogenic perforation during enterogastric tube insertion. The initial diagnostic modality was plain chest radiography with oral water-soluble contrast in three patients. X-ray with contrast showing a feeding tube positioned at the mid-esophagus. Following contrast injection, proximal esophageal dilatation is observed extending to the mid-esophagus, with contrast material visualized within the gastric fundus (Figure 1). Contrast-enhanced X-ray showing contrast material within the mediastinum, likely indicating EP with contrast extravasation into the mediastinal space (Figure 2). In the remaining three patients, no contrast was administered at the time of diagnosis, as clinical and radiological findings were conclusive, and no further advanced diagnostic investigations were performed.

**Figure 3:**
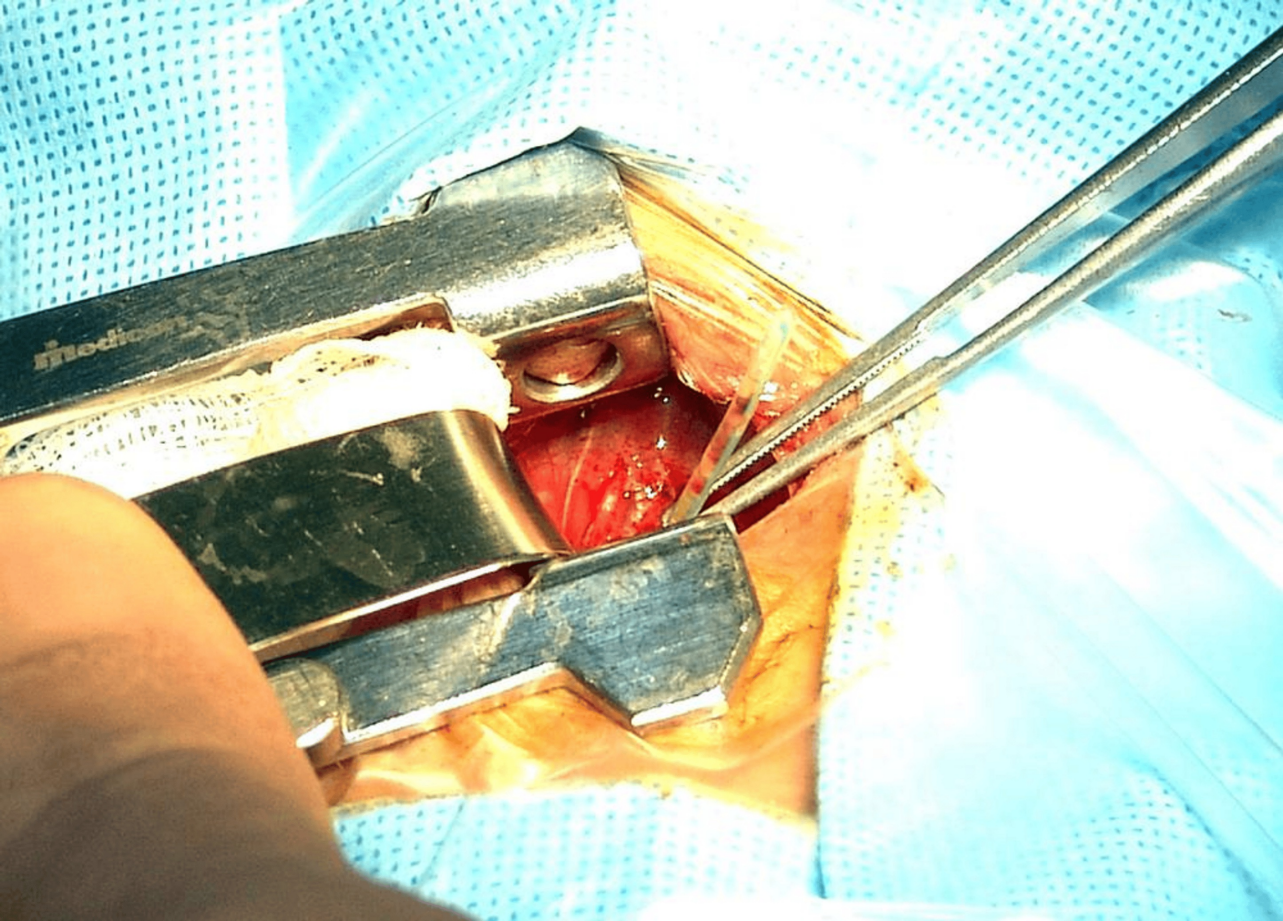
The OGT seen projecting from the esophagus intraoperatively during PDA ligation This figure is an original from the study. OGT: Orogastric tube; PDA: Patent ductus arteriosus

In one patient, the diagnosis of EP was incidental during patent ductus arteriosus (PDA) ligation, where the tip of the OGT was found to be penetrating the esophagus into the pleural cavity then the tip of the feeding tube pulled through the thoracotomy wound (Figure 3). Fluoroscopy was performed after the procedure and revealed no leakage into the mediastinum or the pleural cavity. The perforation was located high in the upper two thirds of the esophagus in four patients and in the lower third of the esophagus in the other three patients.

At the time of diagnosis, three patients exhibited radiological signs of air leakage into the mediastinum accompanied by PT. All patients were kept nil per os (NPO) and received total parenteral nutrition (TPN), with feeding resumed after an average of 22 ± 10 days after serial plain radiograph confirming resolution of pneumomediastinum and PT.

Intravenous antibiotics were initiated for all seven patients at the time of diagnosis. Five patients required intubation and mechanical ventilation due to prematurity; of these, four could not be extubated due to complications related to prematurity, while three were successfully extubated after an average duration of 31 ± 14 days. None of the patients underwent primary thoracic surgical intervention. An overview of patient demographics, gestational age, birth weight, age at diagnosis, initial diagnostic study, findings of radiological images and outcome (Table1). In the patient with intraoperative incidental findings, the OGT was withdrawn to confirm the perforation. The OGT was removed without the need for surgical repair of the esophagus, and the PDA was subsequently ligated successfully.

**Table 1:** Summary of all patients demographics, gestational age, birth weight, age at diagnosis, initial diagnostic study, findings of radiological images, and outcome BPD: Bronchopulmonary dysplasia; TPN: Total parenteral nutrition; PT: Pneumothorax; Amb/slab: Ampicillin sulbactam; Amb/cefo: Ampicillin cefotaxime; LC: Lung collapse; PP: Pneumoperitoneum; High tip: High location in the mediastinum; Low tip: Low location intraabdominal; CXR: Chest X-ray; PDA: Patent ductus arteriosus; BL IVH: Bilateral intraventricular hemorrhage; RDS: Respiratory distress syndrome; PVL: Periventricular leukomalacia; ASD: Atrial septal defect; NEC: Necrotizing enterocolitis; IUGR: Intrauterine growth restriction; DIC: Disseminated intravascular coagulation

Case	Sex	Gestational age (weeks)	Birth weight (grams)	Age at diagnosis (days)	Initial diagnostic study	X-ray findings	Air leak	Parenteral nutrition duration (days)	Sepsis (Y/N)	Antibiotics used	Time to feeding initiation (days)	Mechanical ventilation duration (days)	BPD (Y/N)	Comorbidities	Outcome
1	F	24+4	730	1 D	CXR	Right - PT, high tip	Y	31	Y	Amb/slab	30	45 days	Y	PDA, BL IVH, ambiguous genitalia.	Survived
2	M	27	700	1 D	CXR with contrast	No PT, No fistula, high tip	N	14	N	Amb/cefot	11	No	N	RDS.	Survived
3	M	23	576	2 D	CXR	LC, PT, low tip	Y	92	N	Amb/cefot	22	92	Y	BL IVH, large PDA, severely dilated left atrium and ventricle.	Death, multiorgan failure (secondary to prematurity), cardiovascular arrest
4	F	25	460	3 D	CXR with contrast	Low tip	N	172	Y	Meropenm	32	184	Y	PVL, small ASD, NEC, TPN liver disease, IUGR.	Death, cardiopulmonary failure (secondary to prematurity)
5	F	22+5	520	5 D	CXR	Low tip	N	100	Y	Meropenm	31	100	N	Renal failure, PVL, RDS, large PDA, right PT.	Death, cardiopulmonary failure (secondary to prematurity).
6	M	27	840	1 D	CXR with contrast	High tip, wide mediastinum	Y	49	N	Meropenm	8	17	N	RDS, ASD.	Still admitted
7	F	24	570	7 D	Intr-op day 7	High tip	N	N	Y	Amb/amkikacin/ceffotaxime	10	14	N	Large PDA.	Death, sever fungemia, septic shock, DIC

The comorbidities identified among the patients included sepsis (four patients), bronchopulmonary dysplasia (BPD; three patients), respiratory distress syndrome (RDS; four patients), and congenital cardiac anomalies (six patients). Conservative management of iatrogenic EP was successful in all cases, with enteral feeding initiated without complications. However, four patients ultimately died due to complications related to prematurity following prolonged NICU admission.

## Discussion

In a previous epidemiological study conducted in 2021 involving 861 neonates diagnosed with EP, 77.9% of patients had a birth weight less than 1000 g, and 77.7% were premature (less than 29 weeks of gestation) [7]. In our study, all reviewed patients weighed less than 850 g and were less than 28 weeks of gestation at the time of diagnosis, which reaffirms that prematurity and low birth weight dramatically increase the risk of EP. The thin, underdeveloped esophagus, which is directly related to extreme prematurity and low birth weight, is highly susceptible to perforation during routine procedures, such as enteric tube placement [8].

In the pediatric population, the thoracic esophagus is more prone to injury than the cervical or abdominal esophagus [1,9]. In contrast, EP in neonates is more likely to occur at the pharyngo-esophageal junction proximal to the cricopharyngeal muscle. Two theories were proposed to explain the anatomic site for perforation. First, this site is a normal esophageal stricture, and unsuccessful attempts at enteric tube insertion can result in muscular reflection and blockage of the esophageal lumen; thus, perforation can occur with repetitive attempts. The second theory involves the hyperextension of the neck which can block the esophageal lumen [10,11].

Three of our seven reviewed patients initially sustained EP in the upper part of the esophagus, mimicking esophageal atresia. The presentation of EP is variable and depends on the location of the perforation. EP should be suspected in any infant with difficulties during enteric tube placement, especially when the infant presents with cyanosis, hyperventilation, choking or coughing [12]. EP can mimic esophageal atresia mainly when the perforation is contained within the mediastinum, causing extrinsic compressive force and an obstructive mass that can falsely give the impression of esophageal atresia on radiological image [2]. In comparison, if perforation occurs freely into the pleural cavity, PT or pleural effusion can subsequently occur, and patients may present with acute respiratory distress and rapid clinical deterioration. [2] Difficult enteric tube placement or blood-tinged aspirate from the NGT or OGT is highly suggestive of EP, and the differential diagnostic process should principally include EP in patients at high risk of iatrogenic EP [6]. Two of the included patients had free perforation into the pleural cavity, creating PT, which required chest tube thoracostomy. Initial plain chest radiography is routinely performed after difficult enteric tube placement, as it is a beneficial modality that can help determine the presence of an abnormally located enteric tube tip and subsequent complications of perforation, such as PT, pleural effusion or pneumomediastinum [13]. Nevertheless, if plain films appear normal with high clinical suspicion of EP, contrast studies can be utilized to confirm the diagnosis and the location of perforation [9]. In our case series, two patients underwent chest radiography with water soluble contrast, which confirmed the presence of EP.

In early reported cases of iatrogenic EP, surgical options were prioritized over conservative management [14]. Currently, conservative management has more advantages than operative correction of EP in neonates, as conservative management with NPO, parenteral nutrition and intravenous antibiotics has shown a high rate of success and a shorter time for resuming feeding, along with better overall outcomes [4,15]. When iatrogenic EP in infants was initially described in 1961 by Warden, surgical correction was prioritized, and the patient underwent thoracotomy and primary repair of the esophagus [14]. Over the years, treatment of EP in neonates has shifted to conservative management since it has more favorable outcomes and is less invasive than surgical options [2]. Currently, conservative management that is supportive in nature, including intravenous antibiotics, maintaining NPO status, TPN, and tube thoracostomy, is the first line treatment of EP, as this allows clinicians to treat resulting complications and air leaks [12]. In our case series, conservative management was chosen for all the patients in this study. TPN with NPO status was initiated at diagnosis for all the reviewed patients, and feeding resumed after an average period of 22 days. In our experience, conservative supportive management showed satisfactory results without subjecting the patient to invasive procedures that may not be tolerable in patients in poor condition with multiple comorbidities. EP alone as a single factor does not increase mortality, as low birth weight and extreme prematurity strongly contribute to mortality. In our series, three patients died due to unfortunate sequelae of prematurity, such as cardiopulmonary arrest or multiple organ failure, but during their NICU admission, feeding was resumed and was tolerated well, demonstrating the success of conservative management.

Study limitations

This single-center study was limited in scope, as it did not include patients with iatrogenic EP beyond the gestational age of 27 weeks or those with a birth weight greater than 1,000 grams. The small sample size and narrow inclusion criteria further restrict the generalizability of the findings to broader neonatal populations

## Conclusions

Early recognition of iatrogenic EP is crucial for improving outcomes in extremely premature babies with low birth weigh. Clinical suspicion should remain high in the presence of feeding difficulties and respiratory distress in neonates following enterogastric tube insertion. Iatrogenic EP diagnosis can be difficult in extremely premature babies with low birth weight but non-operative management of such cases can be considered a safe efficient option with good outcomes. Extreme prematurity and low birth weight are risk factors for iatrogenic EP. Despite successful management of the perforation itself, overall outcomes are strongly influenced by the underlying prematurity and associated comorbidities. Multidisciplinary care involving neonatology, radiology, and surgery teams is essential for optimizing outcomes in these fragile patients.
